# Supplementary material for: Stratifin as a novel diagnostic biomarker in serum for diffuse alveolar damage
Source: Nat Commun. 2022 Oct 4;13:5854. doi: 10.1038/s41467-022-33160-9 (PMC9532442; doi:10.1038/s41467-022-33160-9)
Supplement: Supplementary file 1 — Supplementary Information [file 41467_2022_33160_MOESM1_ESM.pdf]

Supplementary Figures

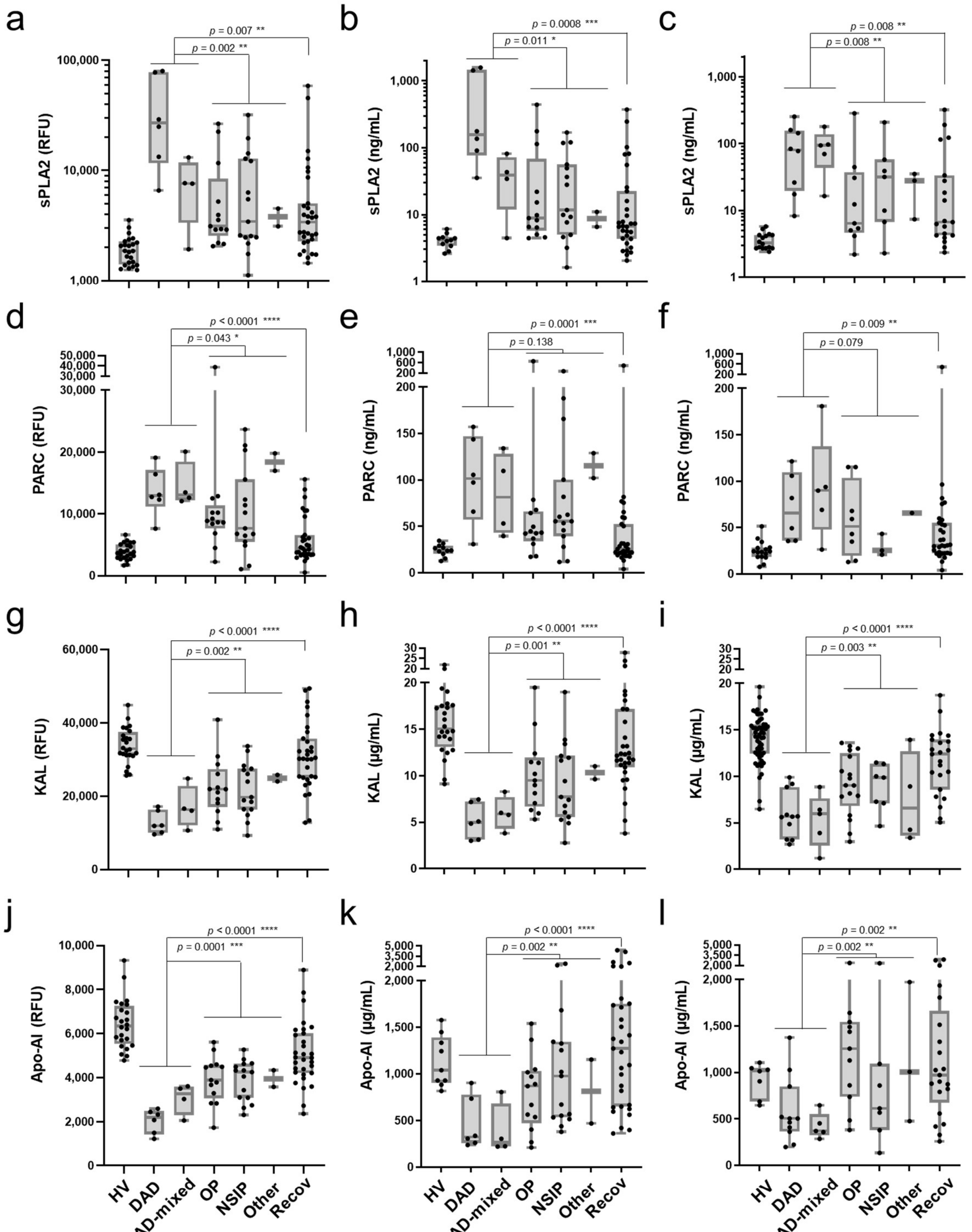

m

		AUC (95% CI)			
		sPLA2	PARC	Apo-AI	KAL
DAD group vs HV	Discovery	0.97 (0.91 - 1.0)	0.98 (0.94 - 1.0)	0.98 (0.92 - 1.0)	1.0 (1.0 - 1.0)
	Validation	1.0 (1.0 - 1.0)	0.94 (0.86 - 1.0)	0.88 (0.74 - 1.0)	0.99 (0.97 - 1.0)
DAD group vs Recovery	Discovery	0.84 (0.69 - 0.98)	0.88 (0.77 - 0.99)	0.91 (0.80 - 1.0)	0.95 (0.88 - 1.0)
	Validation	0.79 (0.63 - 0.95)	0.81 (0.64 - 0.98)	0.81 (0.67 - 0.95)	0.90 (0.81 - 1.0)
DAD group vs non-DAD	Discovery	0.77 (0.59 - 0.95)	0.66 (0.47 - 0.85)	0.82 (0.67 - 0.97)	0.83 (0.70 - 0.96)
	Validation	0.79 (0.62 - 0.95)	0.73 (0.52 - 0.94)	0.80 (0.66 - 0.94)	0.77 (0.63 - 0.90)

Supplementary Figure 1. Blood levels of sPLA2, PARC, KAL and Apo-AI in identifying patients with acute DILD. SOMAscan signals (**a, d, g, j**) and ELISA data (**b, c, e, f, h, i, k, l**) of sPLA2 (**a-c**), PARC (**d-f**), KAL (**g-i**), and Apo-AI (**j-l**) for the healthy volunteer (HV) and DILD patients in the Discovery cohort (**a, b, d, e, g, h, j, k**) and Validation cohort (**c, f, i, l**). The boxes indicate interquartile ranges (75% and 25%) and medians; whiskers show min and max values. Differences between the two groups were compared by a two-tailed Mann-Whitney U-test (unadjusted): * $p < 0.05$; ** $p < 0.01$; *** $p < 0.001$; **** $p < 0.0001$. DAD: diffuse alveolar damage; OP: organizing pneumonia; NSIP: nonspecific interstitial pneumonia; Recov: all recovery patients. **h.** The area under the curve (AUC) was derived from receiver operating characteristic (ROC) curves, and the 95% confidence intervals (95%CIs) were determined for the biomarkers in comparative analyses. Numbers of samples (**a, d, g, j**): HV (n = 24), DAD (n = 6), DAD-mixed (n = 4), OP (n = 13), NSIP (n = 15), other (n = 2), Recov (n = 31). Numbers of samples for the remaining groups and corresponding median values (range) are shown in Supplementary Table 7. Source data are provided as a Source data file.

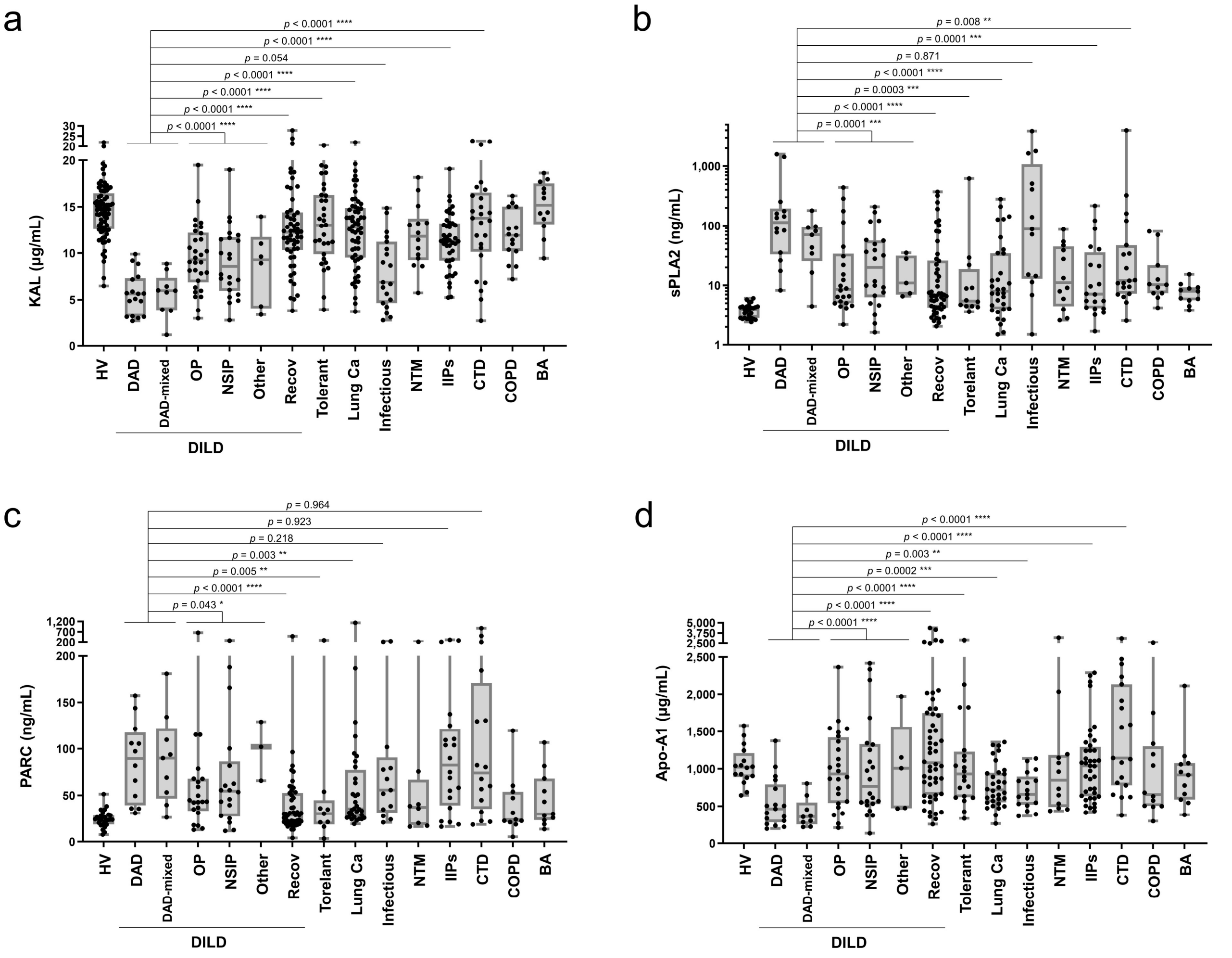

Supplementary Figure 2. Blood levels of KAL, sPLA2, PARC and Apo-AI in healthy volunteers and patients with various lung diseases. Results for the Combined cohort are shown. Serum levels of KAL (**a**) and plasma levels of sPLA2 (**b**), PARC (**c**), and Apo-AI (d) were measured by ELISA. The boxes indicate interquartile ranges (75% and 25%) and medians; whiskers show min and max values. Differences between the two groups were compared by a two-tailed Mann-Whitney U-test (unadjusted): *$p < 0.05$; ** $p < 0.01$; *** $p < 0.001$; **** $p < 0.0001$. DAD: diffuse alveolar damage; OP: organizing pneumonia; NSIP: nonspecific interstitial pneumonia; Recov: all DILD patients in recovery; Tolerant: tolerant control; Lung Ca: lung cancer; IIPs: idiopathic interstitial pneumonia; CTD: lung disease associated with connective tissue disease; COPD: chronic obstructive pulmonary disease; NTM: nontuberculous mycobacteria; BA: bronchial asthma; infectious: bacterial and mycotic pneumonia. Numbers of samples and corresponding median values (range) for each group are shown in Supplementary Table 9. Source data are provided as a Source data file.

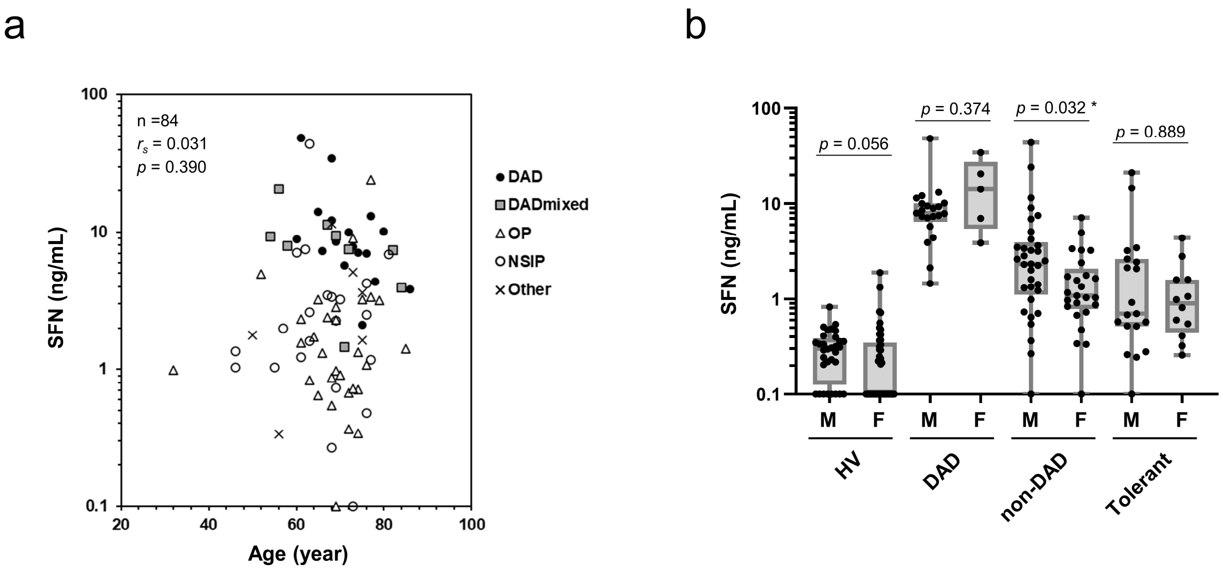

Supplementary Figure 3. Correlations between serum SFN levels and demographics. Data of the Combined set are shown. **a** The correlation between serum SFN levels and the ages of the acute DILD patients. The correlation coefficients r_s and its p value was calculated by Spearman's correlation analysis. **b** Distribution of serum SFN levels by sex of healthy volunteers (HV), acute DILD patients and tolerant controls. Numbers of samples: HV (M: n = 32, F: n = 45), DAD (M: n = 21, F : n = 5), non-DAD (M: n = 33, F: n = 25), tolerant controls (M: n = 19, F: n = 12). The boxes indicate interquartile ranges (75% and 25%) and medians; whiskers show min and max values. Differences between the two groups were compared by a two-tailed Mann-Whitney U-test (unadjusted): *$p < 0.05$. M: male; F: female; DAD: diffuse alveolar damage; DAD group: DAD + DAD mixed pattern. Source data are provided as a Source data file.

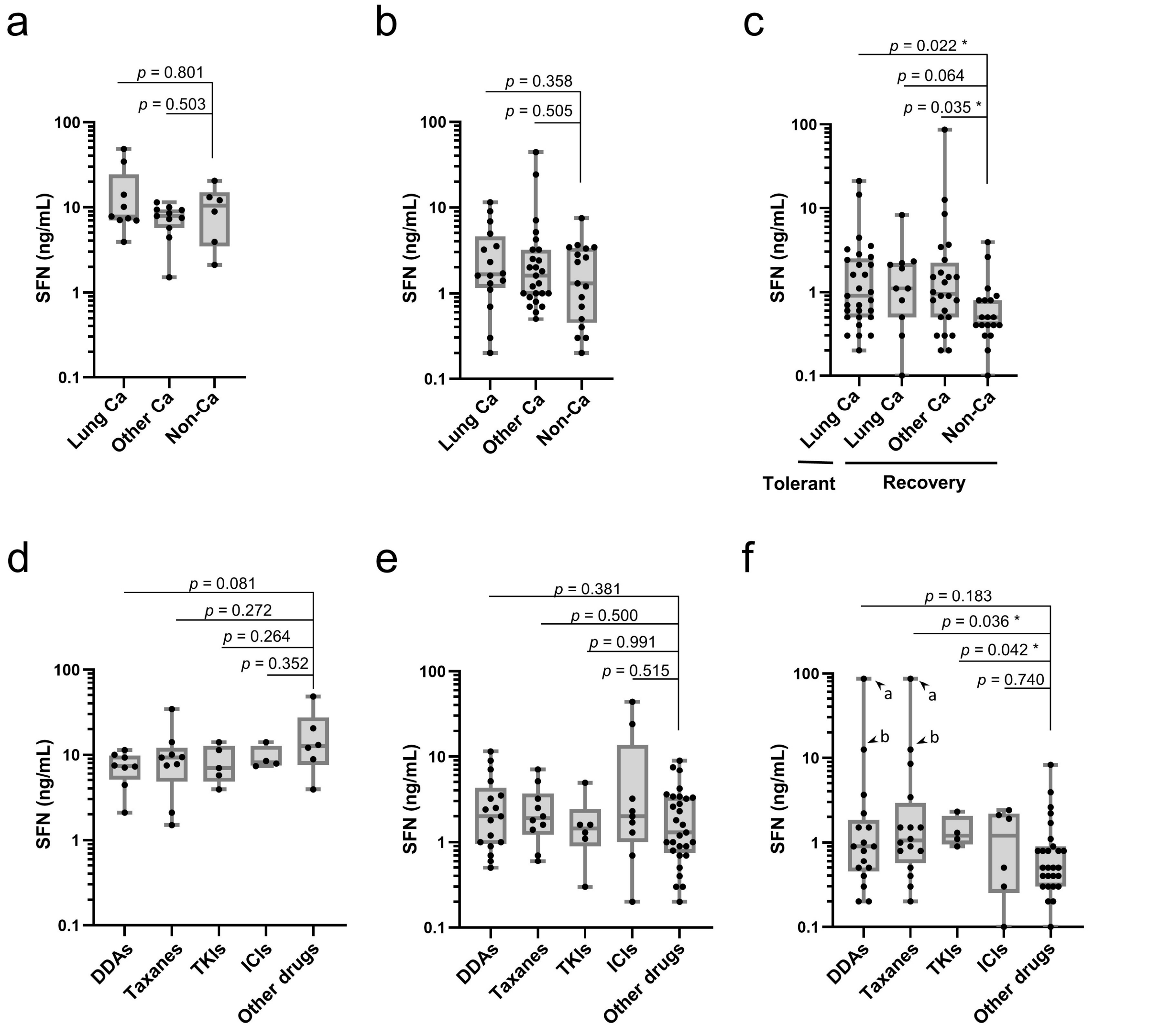

Supplementary Figure 4. Distribution of serum SFN levels by underlying disease and medication in DILD-onset patients and tolerant control patients. Data of the Combined set are shown. Acute- and recovery-phase DILD patients as well as tolerant control patients were categorized by underlying disease (**a, b, c**) and causative drug (**d, e, f**). The SFN levels in each category were compared using box-plot graphs. **a, d.** Acute-stage patients in the DAD group (DAD + DAD-mixed pattern). **b, e.** Acute-stage patients in the non-DAD group. **c.** Recovery-phase DILD patients and tolerant controls. **f.** Recovery-phase DILD patients. Numbers of samples: n = 9, 11, 6 (**a**), n = 16, 25, 17 (**b**), n = 29, 11, 24, 19 (**c**), n = 8, 9, 5, 4, 6 (**d**), n = 17, 10, 6, 9, 29 (**e**), and n = 17, 16, 4, 6, 27 (**f**) for each box from left to right. The boxes indicate interquartile ranges (75% and 25%) and medians; whiskers show min and max values. Differences between the two groups were compared by a two-tailed Mann-Whitney U-test (unadjusted): *$p < 0.05$. Lung Ca: lung cancer; Other Ca: other tissue cancers (e.g., pancreatic cancer, breast cancer, bladder cancer, and colon cancer); Non-Ca: non-cancer conditions (e.g., heart failure, rheumatoid arthritis, and infectious

disease). DDAs: DNA-damaging agents (e.g., gemcitabine, platinum-based drugs, and bleomycin); taxane: taxanes (paclitaxel, and docetaxel); TKIs: tyrosine kinase inhibitors (e.g., erlotinib, osimertinib, axitinib, and bevacizumab); ICIs: immune checkpoint inhibitors (e.g., nivolumab and pembrolizumab); and other drugs (e.g., mTOR inhibitors as well as antibiotics, anti-rheumatoid drugs, antiarrhythmic agents, and Chinese herbal medicines). In Graph **f**, the arrows “a” and “b” indicate the same patient, who received both DDA and taxane. DAD: diffuse alveolar damage; OP: organizing pneumonia; NSIP: nonspecific interstitial pneumonia. Source data are provided as a Source data file.

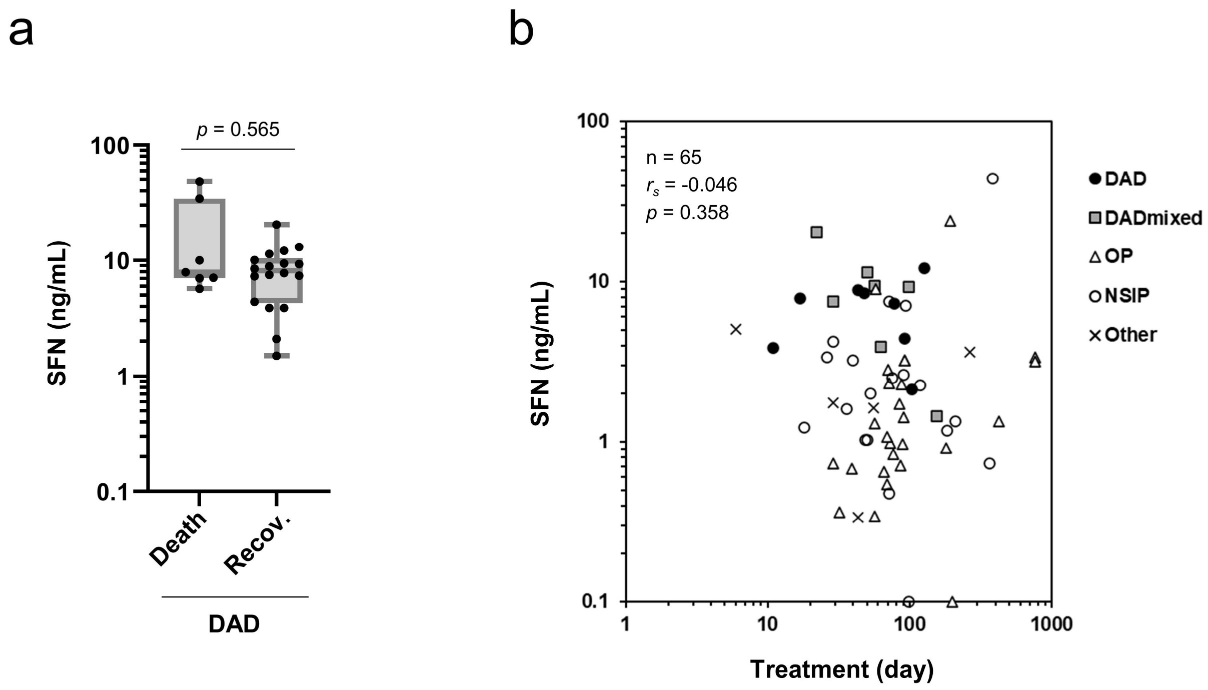

Supplementary Figure 5. Correlations between serum SFN levels and outcomes of DILD patients. Data of the Combined set are shown. **a** Comparison of serum SFN levels in the death cases (due to DILD, n = 7) and the recovered cases (n = 18) in the DAD group. There were no death cases in the non-DILD patient group. The boxes indicate interquartile ranges (75% and 25%) and medians; whiskers show min and max values. Differences between the groups was compared by a two-tailed Mann-Whitney U-test. **b** The correlation between serum SFN levels at acute phase and the numbers of days required for DILD treatment. The correlation coefficients r_s and its p value were calculated by Spearman's correlation analysis. DAD group: DAD + DAD mixed pattern. ns, not significant. DAD: diffuse alveolar damage; OP: organizing pneumonia; NSIP: nonspecific interstitial pneumonia. Source data are provided as a Source data file.

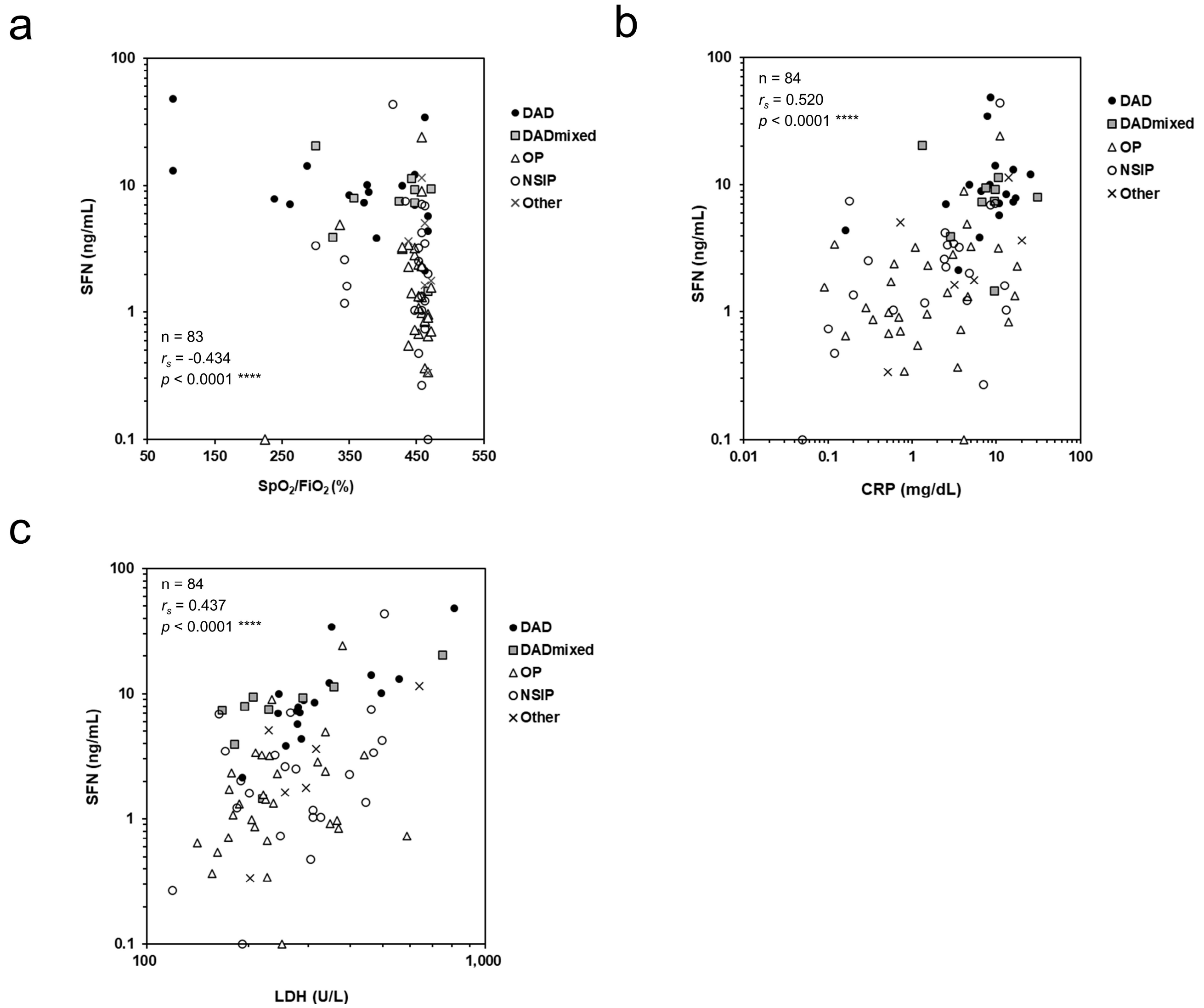

Supplementary Figure 6. Correlation between the serum levels of SFN and the pulmonary function and inflammation parameters. Data of the Combined set are shown. The correlations between the levels of SFN and pulmonary function parameters and clinical chemistry values during the acute stage of drug-induced interstitial pneumonia were assessed (**a**) *versus* the ratio of percutaneous arterial oxygen saturation to fractional inspired oxygen (SpO_2/FiO_2), (**b)** *versus* the levels of LDH, and (**c**) *versus* the levels of CRP. The values of the correlation coefficients r_s and p were calculated as per Spearman's correlation analysis. DAD: diffuse alveolar damage; OP: organizing pneumonia; NSIP: nonspecific interstitial pneumonia. Source data are provided as a Source data file.

Supplementary Table 1. Collected laboratory test values of analyzed patients.

White blood cells								
**Patient group**			**WBC (×10^3/µL) [*n*]**	**Lymphocyte (%) [*n*]**	**Neutrophile (%) [*n*]**	**Eosinophile (%) [*n*]**	**Monocyte (%) [*n*]**	**Basophile (%) [*n*]**
Discovery cohort	DILD acute	**DAD group**	**7.3 (2.6 - 23.7) [10]**	**12.5 (4.1 - 35.4) [10]**	**80.4 (48.4 - 93.6) [10]**	**1.1 (0 - 8.1) [10]**	**6.1 (2.3 - 11) [10]**	**0.3 (0 - 1.5) [10]**
		DAD	9.8 (3.6 - 23.7) [6]	8.6 (4.1 - 18.6) [6]	83.8 (75.2 - 93.6) [6]	0.9 (0 - 3.1) [6]	4.9 (2.3 - 7.5) [6]	0.2 (0 - 1) [6]
		DAD-mixed	5.8 (2.6 - 8.4) [4]	28.1 (15.2 - 35.4) [4]	61 (48.4 - 78.1) [4]	1.1 (0 - 8.1) [4]	7.6 (5.4 - 11) [4]	0.4 (0.2 - 1.5) [4]
		**non-DAD group**	**6.6 (2.2 - 12.7) [30]**	**17.5 (2.5 - 42) [30]**	**71.5 (16 - 92) [30]**	**2.3 (0 - 19) [30]**	**7.1 (1 - 21.5) [30]**	**0.4 (0 - 1.2) [30]** [ns, #]
		OP	7.8 (2.2 - 12.2) [13]	22.1 (2.5 - 35.1) [13]	66.9 (53.1 - 78.5) [13]	2.9 (0.6 - 13) [13]	7.4 (5.1 - 12.9) [13]	0.5 (0 - 1) [13]
		NSIP	6.5 (3.8 - 10.6) [15]	15.5 (5.2 - 42) [15]	73.1 (16 - 88.9) [15]	1.4 (0.2 - 19) [15]	7.2 (2.7 - 21.5) [15]	0.3 (0 - 1.2) [15]
		Other	8.6 (4.5 - 12.7) [2]	7.5 (6 - 9) [2]	86 (80 - 92) [2]	1 (0 - 2) [2]	3 (1 - 5) [2]	0 (0 - 0) [2]
	DILD recovery	**All**	**5.4 (2.9 - 35.4) [31]**	**23 (5.1 - 50.2) [31]**	**70.2 (39.4 - 93.6) [31]**	**1.3 (0 - 9.8) [31]**	**6.4 (0.9 - 12.9) [31]**	**0.2 (0 - 1) [31]** [ns, #]
		KW test	ns	ns	ns	ns	ns	$p = 0.045$ *
Validation cohort	DILD acute	**DAD group**	**7.3 (3.3 - 105.1) [16]**	**15.7 (5.6 - 33.4) [16]**	**75 (42 - 88.8) [16]**	**1 (0 - 6) [16]**	**7.2 (0 - 28) [16]**	**0.3 (0 - 2.5) [16]**
		DAD	5.9 (3.3 - 105.1) [11]	15.8 (6.6 - 33.4) [11]	75.2 (42 - 86.5) [11]	1.0 (0 - 6) [11]	7.3 (0 - 19) [11]	0.3 (0 - 0.7) [11]
		DAD-mixed	8.5 (3.9 - 15.7) [5]	12 (5.6 - 18.1) [5]	70.9 (55.5 - 88.8) [5]	1.5 (0.5 - 5.9) [5]	7.1 (4.7 - 28) [5]	0.5 (0.1 - 2.5) [5]
		**non-DAD group**	**6.5 (3.4 - 11.3) [28]**	**18.3 (4.7 - 45.7) [28]**	**69 (41 - 87.3) [28]**	**2.1 (0 - 27.2) [28]**	**7.4 (3.3 - 13.6) [28]** [ns, #]	**0.3 (0 - 1.2) [28]**
		OP	5.9 (3.4 - 10.3) [17]	18.4 (4.7 - 42.9) [17]	68.9 (47.7 - 86.4) [17]	2 (0 - 10.7) [17]	7.4 (3.5 - 13.6) [17]	0.3 (0 - 1.2) [17]
		NSIP	7.3 (4.4 - 11.3) [7]	19 (10.8 - 45.7) [7]	64.5 (41 - 81.8) [7]	1.7 (0.5 - 25) [7]	7.1 (4.3 - 11.5) [7]	0.2 (0.1 - 1) [7]
		Other	7.6 (4.8 - 9.5) [4]	17.6 (6.1 - 22.9) [4]	71.2 (46 - 87.3) [4]	2.6 (0.8 - 27.2) [4]	6.1 (3.3 - 9) [4]	0.4 (0 - 1) [4]
	DILD recovery	**All**	**7.4 (0 - 35.7) [23]**	**18.5 (3 - 49.9) [22]**	**70.5 (46.2 - 92.6) [22]**	**0.9 (0 - 9.6) [22]**	**5.7 (1.8 - 10.5) [22]** [ns, #]	**0.2 (0 - 2) [22]**
		KW test	ns	ns	ns	ns	$p = 0.040$ *	ns
Tolerant controls	Tolerant controls		6 (2.9 - 9.8) [31] [ns, #]	22 (8.4 - 44.9) [31] [ns, #]	67.9 (39 - 87) [31] [ns, #]	1.9 (0 - 14.8) [31] [ns, #]	6.3 (1.4 - 19) [31] [ns, #]	0.5 (0 - 4) [31]
Disease controls	Lung cancer		6.7 (3.2 - 23.1) [58] [ns, #]	18.5 (3.6 - 69.9) [57] [ns, #]	68.6 (21.2 - 95) [57] [ns, #]	2.4 (0 - 22.6) [57] [ns, #]	5.9 (1.4 - 14.4) [57] [ns, #]	0.4 (0 - 1.6) [57]
	Infectious		7.9 (4.1 - 42.2) [19] [ns, #]	13.6 (3 - 41.3) [19] [ns, #]	72.5 (44.5 - 92.5) [19] [ns, #]	1.9 (0 - 7.5) [19] [ns, #]	6.9 (4.5 - 12) [19] [ns, #]	0.3 (0 - 2) [19]
	NTM		6.1 (3.3 - 13.5) [12] [ns, #]	22.4 (11.4 - 34.1) [12] [ns, #]	65.1 (51.3 - 82.8) [12] [ns, #]	3.1 (0 - 13.6) [12] [ns, #]	8.4 (3.4 - 11) [12] [ns, #]	0.4 (0 - 2.4) [12]
	IIPs		7.9 (4 - 21.4) [43] [ns, #]	22.9 (4 - 53.8) [43] [*, a, #]	69 (31.2 – 87.1) [43] [ns, #]	2.4 (0 – 23.9) [43] [ns, #]	6 (0 – 12.8) [43] [ns, #]	0.5 (0 – 1.3) [43]
	CTD		9.1 (4.2 – 13.9) [25] [ns, #]	21.4 (6.5 – 35.6) [25] [ns, #]	70.6 (26 – 89.4) [25] [ns, #]	1.2 (0.2 – 3.9) [25] [ns, #]	4.6 (2.2 – 9.2) [25] [*, e, #]	0.3 (0 - 0.8) [25]
	COPD		7.1 (2.8 - 11.6) [15] [ns, #]	17.1 (4 - 30.6) [14] [ns, #]	72.4 (63.4 - 88) [14] [ns, #]	2 (0 - 6.5) [14] [ns, #]	6 (3 - 8.5) [14] [ns, #]	0.4 (0 - 1) [14]

	BA		7.2 (4.7 - 11.2) [11] $^{ns, \#}$	26 (8.4 - 47.6) [11] **$^{, b, \#}$	60.3 (37.5 - 87.5) [11] **$^{, c, \#}$	6.6 (0 - 11.9) [11] ***$^{, d, \#}$	4.7 (3.7 - 7.6) [11] $^{ns, \#}$	0.3 (0 - 1.1) [11]
Combined DAD group			7.3 (2.6 - 105.1) [26]	15.4 (4.1 - 35.4) [26]	76.5 (42 - 93.6) [26]	1 (0 - 8.1) [26]	7 (0 - 28) [26]	0.3 (0 - 2.5) [26]
		KW test	p = 0.0008 ***	p = 0.006 **	p = 0.008 **	p = 0.002 **	p = 0.004 **	ns
**Respiratory and other parameters**								
**Patient group**			**P/F ratio (mmHg) [*n*]**	**SpO_2 (%) [*n*]**	**CRP (mg/dL) [*n*]**	**LDH (U/L) [*n*]**	**Creatinine (mg/dL) [*n*]**	**BNP (pg/mL) [*n*]**
Discovery cohort	DILD acute	**DAD group**	**241.4 (113 - 377.1) [7]**	**94 (50 - 99) [10]**	**9.1 (1.3 - 25.3) [10]**	**280 (167 - 812) [10]**	**0.8 (0.7 - 2) [7]**	**78.6 (20.2 - 225) [6]**
		DAD	241.4 (113 - 377.1) [5]	85 (50 - 98) [6]	13.3 (6.4 - 25.3) [6]	313.5 (258 - 812) [6]	1 (0.7 - 1.5) [4]	76.2 (20.2 - 225) [4]
		DAD-mixed	277.5 (235 - 320) [2]	97 (94 - 99) [4]	7.1 (1.3 - 9.5) [4]	213 (167 - 751) [4]	0.8 (0.7 - 2) [3]	109.1 (27.1 - 191) [2]
		**non-DAD group**	**375.7 (247.6 - 561.9) [12] **$^{, f, \#}$**	**96 (91 -99) [29]**	**2.6 (0.1 - 17.7) [30] *$^{, g, \#}$**	**234 (141 - 468) [30]**	**0.9 (0.4 - 1.2) [15]**	**31.2 (7.3 - 447) [23]**
		OP	407.1 (327.6 - 561.9) [5]	96.5 (92 - 99) [12]	1.2 (0.2 - 17.7) [13]	204 (141 - 369) [13]	0.9 (0.5 - 1.1) [5]	20.1 (9.5 - 106) [10]
		NSIP	368.6 (254.8 - 428.1) [5]	96 (91 - 98) [15]	2.6 (0.1 - 13) [15]	266 (164 - 468) [15]	0.8 (0.4 - 1.2) [10]	48.8 (8.7 - 447) [12]
		Other	315.2 (247.6 - 382.9) [2]	98 (97 - 99) [2]	3.1 (0.7 - 5.4) [2]	262.5 (229 - 296) [2]	n/a	7.3 [1]
	DILD recovery	**All**	**348.6 [1]**	**97 (94 - 99) [29]**	**0.6 (0 - 19.3) [30] ***$^{, h, \#}$**	**239 (146 - 3356) [31]**	**0.9 (0.3 - 1.8) [15]**	**106.9 (8.2 - 584) [8]**
		KW test	p = 0.006 **	ns	p = 0.0002 ***	ns	ns	ns
Validation cohort	DILD acute	**DAD group**	**287.1 (50 - 482.1) [9]**	**93.5 (78 - 100) [16]**	**8.9 (0.2 - 31) [16]**	**285.5 (182 - 495) [16]**	**0.8 (0.4 - 1.4) [11]**	**51.4 (10.9 - 304) [11]**
		DAD	282.4 (50 - 336.1) [8]	94 (78 - 98) [11]	7.8 (0.2 - 16) [11]	287 (192 - 495) [11]	0.7 (0.4 - 1.2) [9]	57.2 (15.6 - 304) [7]
		DAD-mixed	482.1 [1]	93 (89 - 100) [5]	9.8 (2.9 - 31) [5]	230 (182 - 358) [5]	1.1 (0.8 - 1.4) [2]	24.2 (10.9 - 76) [4]
		**non-DAD group**	**342.1 (168 - 600) [12]**	**95.5 (87 -99) [28]**	**2.8 (0.1 - 20) [28] *$^{, i, \#}$**	**253.5 (119 - 640) [28]**	**0.8 (0.4 - 1.6) [19]**	**31.4 (7 - 911) [17]**
		OP	342.1 (168 - 600) [10]	95 (90 - 99) [17]	1.6 (0.1 - 16.5) [17]	237 (178 - 589) [17]	0.8 (0.4 - 1.4) [13]	31.4 (7 - 911) [9]
		NSIP	435.7 (435.7 - 435.7) [1]	96 (87 - 98) [7]	2.4 (0.1 - 11) [7]	306 (119 - 505) [7]	0.8 (0.4 - 1.6) [4]	107.5 (8.6 - 306) [4]
		Other	210.8 (210.8 - 210.8) [1]	96.5 (92 - 98) [4]	8.6 (0.5 - 20) [4]	286 (202 - 640) [4]	0.9 (0.8 - 1) [2]	24.8 (11.9 - 207) [4]
	DILD recovery	**All**	**325.7 (270.5 - 381) [2]**	**95.5 (89 - 99) [20]**	**0.4 (0 - 28.8) [22] ***$^{, h,\#}$**	**227.5 (152 - 439) [22]**	**1.1 (0.4 - 1.2) [9]**	**n/a**
		KW test	ns	ns	p = 0.0004 ***	ns	ns	ns
Tolerant controls	Tolerant controls		n/a	97 (95 - 99) [31] ****$^{, j, \#}$	0.1 (0 - 16.6) [30] ****$^{, j, \#}$	217 (25 - 442) [31] *$^{, o, \#}$	0.9 (0.5 - 1.3) [24]	n/a
Disease controls	Lung cancer		n/a	96 (90 - 99) [56] **$^{, b, \#}$	0.9 (0 - 24.6) [55] ****$^{, j, \#}$	224 (2.9 - 1386) [58] *$^{, p, \#}$	0.8 (0.4 - 5.9) [50]	n/a
	Infectious		n/a	96 (91 - 99) [17] $^{ns, \#}$	4.7 (0.1 - 107) [18] $^{ns, \#}$	193 (16 - 442) [19] **$^{, q, \#}$	0.8 (0.4 - 1.5) [17]	n/a

	NTM		n/a	97 (36 - 98) [12] $^{ns, \#}$	0.6 (0.1 - 10.7) [12] $^{**, m, \#}$	188 (143 - 294) [12] $^{**, q, \#}$	0.7 (0.5 - 1.1) [10]	n/a
	IIPs		n/a	95 (90 - 98) [43] $^{ns, \#}$	0.5 (0 - 13.2) [43] $^{****, j, \#}$	268 (102 - 543) [43] $^{ns, \#}$	0.8 (0.5 - 2) [38]	n/a
	CTD		n/a	96 (93 - 99) [25] $^{*, k, \#}$	0.2 (0 - 18.2) [25] $^{****, j, \#}$	237.5 (171 - 510) [24] $^{ns, \#}$	0.7 (0.5 - 1.7) [20]	n/a
	COPD		n/a	94 (90 - 98) [15] $^{ns, \#}$	1.1 (0 - 5.2) [15] $^{***, n, \#}$	255 (151 - 3260) [15] $^{ns, \#}$	0.8 (0.4 - 1.8) [14]	n/a
	BA		n/a	98 (95 - 99) [10] $^{**, l, \#}$	0.1 (0 - 1) [10] $^{****, j, \#}$	194 (132 - 339) [11] $^{*, r, \#}$	0.8 (0.5 - 1.1) [9]	n/a
Combined DAD group			267.3 (50 - 482.1) [16]	94 (50 - 100) [26]	9.1 (0.2 - 31) [26]	282.5 (167 - 812) [26]	0.8 (0.4 - 2) [18]	51.4 (10.9 - 304) [17]
		KW test	n/a	p <0.0001 ****	p <0.0001 ****	p <0.0001 ****	ns	n/a

Median values (ranges) are shown; [n]: number of patients analyzed. Differences among the groups were first tested using Kruskal-Wallis (KW) test. In the Discovery and Validation cohorts, healthy volunteers, the DAD group and the non-DAD group were compared (comparison a). The tolerant and disease controls were compared with the combined data of the DAD group in the Discovery and Validation cohorts (comparison b). When significant differences were observed in the KW test, then Dunn's test was performed to compare the DAD group with healthy volunteers and the non-DAD group (comparison a) or to compare the combined DAD group with tolerant and disease controls (comparison b). $^{\#}$Differences to the DAD group or combined DAD group were assessed by Dunn's test. *p < 0.05; **p < 0.01; ***p < 0.001; ****p < 0.0001; ns, not significant. HV: healthy volunteer; DAD: diffuse alveolar damage; OP: organizing pneumonia; NSIP: nonspecific interstitial pneumonia; IIPs: idiopathic interstitial pneumonias; CTD: lung disease associated with connective tissue disease; COPD: chronic obstructive pulmonary disease; NTM: nontuberculous mycobacteria; BA: bronchial asthma; infection: bacterial and mycotic pneumonia; n/a: not available.

^{a}p = 0.042 compared with the combined DAD group.

^{b}p = 0.006 compared with the combined DAD group.

^{c}p = 0.005 compared with the combined DAD group.

^{d}p = 0.0008 compared with the combined DAD group.

^{e}p = 0.025 compared with the combined DAD group.

^{f}p = 0.009 compared with the DAD group (Discovery cohort).

[g]$p = 0.018$ compared with the DAD group (Discovery cohort).
[h]$p = 0.0001$ compared with the DAD group (Discovery or Validation cohorts).
[i]$p = 0.049$ compared with the DAD group (Validation cohort).
[j]$p < 0.0001$ compared with the combined DAD group.
[k]$p = 0.013$ compared with the combined DAD group.
[l]$p = 0.003$ compared with the combined DAD group.
[m]$p = 0.008$ compared with the combined DAD group.
[n]$p = 0.0006$ compared with the combined DAD group.
[o]$p = 0.021$ compared with the combined DAD group.
[p]$p = 0.034$ compared with the combined DAD group.
[q]$p = 0.002$ compared with the combined DAD group.
[r]$p = 0.023$ compared with the combined DAD group.

Supplementary Table 2. Underlying diseases in DILD-onset and tolerant control patients.

Patient group [*n*]			Lung cancer (Ad / Sq / SC / other type)		Other tissue cancers (PaCa / EsCa / BrCa / other Ca)		Non-cancer diseases (heart / RA / Inf / other disease)	
Discovery cohort	DILD acute [40]		10	(6/0/1/3)	19	(7/2/4/6)	11	(5/1/1/4)
		**DAD group [10]**	**4 (40%)**	**(3/0/0/1)**	**3 (15.8%)**	**(0/2/0/1)**	**3 (27.3%)**	**(0/1/1/1)**
		DAD [6]	3	(2/0/0/1)	1	(0/0/0/1)	2	(0/0/1/1)
		DAD-mixed [4]	1	(1/0/0/0)	2	(0/2/0/0)	1	(0/1/0/0)
		**non-DAD group [30]**	**6 (60%)**	**(3/0/1/2)**	**16 (84.2%)**	**(7/0/4/5)**	**8 (72.7%)**	**(5/0/0/3)**
		OP [13]	5	(3/0/0/2)	5	(1/0/1/3)	3	(2/0/0/1)
		NSIP [15]	1	(0/0/1/0)	9	(5/0/2/2)	5	(3/0/0/2)
		Other [2]	0		2	(1/0/1/0)	0	
	DILD recovery [31]	All	5	(3/0/0/2)	16	(7/2/4/3)	10	(5/1/1/3)
Validation cohort	DILD acute [45]		15	(9/3/3/0)	17	(3/1/0/13)	12	(2/3/0/7)
		**DAD group [16]**	**5 (33.3%)**		**8 (47.1%)**		**3 (25%)**	
		DAD [11]	5	(2/3/0/0)	4	(1/0/0/3)	2	(0/1/0/1)
		DAD-mixed [5]	0		4	(2/1/0/1)	1	(0/0/1/0)
		**non-DAD group [28]**	**10 (66.7%)**		**9 (52.9%)**		**9 (75%)**	
		OP [17]	7	(6/0/1/0)	6	(0/0/0/6)	4	(1/1/0/2)
		NSIP [7]	1	(0/0/1/0)	3	(0/0/0/3)	3	(1/1/0/1)
		Other [4]	2	(1/0/1/0)	0		2	(0/0/0/2)
	DILD recovery [23]	All	6	(5/0/1/0)	8	(2/1/0/5)	9	(3/0/1/5)
Tolerant controls [31]			29	(25/2/1/1)	1	(0/0/0/1)	1	(0/0/0/1)

Data of the DAD and non-DAD groups are shown in bold. The types of lung cancer were categorized as follows: adenocarcinoma (Ad), squamous cell carcinoma (Sq), small-cell carcinoma (SC), and other lung cancer, which included non-small cell carcinoma and pleomorphic carcinoma. Cancers of other tissues were categorized as follows: pancreatic cancer (PaCa), esophageal cancer (EsCa), breast cancer (BrCa), and other cancers including kidney cancer, colon cancer, and bladder cancer (other Ca). Most cases of cancers of other tissues were PaCa, EsCa, or BrCa. Additionally, non-cancer conditions were categorized as follows: heart disease (heart), rheumatoid arthritis (RA), infectious disease (Inf), and other diseases (other diseases). [*n*]: number of patients analyzed. DAD: diffuse alveolar damage; OP: organizing pneumonia; NSIP: nonspecific interstitial pneumonia.

Supplementary Table 3. Suspected causal drug types in DILD-onset patients and medications administered to tolerant control patients.

Patient group			DDAs	Taxanes	TKIs	ICIs	Other drugs
Discovery cohort	DILD acute		12	12	4	5	18
		**DAD group**	**0 (0%)**	**3 (25%)**	**2 (50%)**	**1 (20%)**	**4 (22.2%)**
		DAD	0	1	2	0	3
		DAD-mixed	0	2	0	1	1
		**non-DAD group**	**12 (100%)**	**9 (75%)**	**2 (50%)**	**4 (80%)**	**14 (77.8%)**
		OP	4	3	1	3	6
		NSIP	6	4	1	1	7
		Other	2	2	0	0	1
	DILD recovery	All	11	11	2	3	14
Validation cohort	DILD acute		13	7	7	8	17
		**DAD group**	**8 (61.5%)**	**6 (85.7%)**	**3 (42.9%)**	**3 (37.5%)**	**2 (11.8%)**
		DAD	5	4	2	2	1
		DAD-mixed	3	2	1	1	1
		**non-DAD group**	**5 (38.5%)**	**1 (14.3%)**	**4 (57.1%)**	**5 (62.5%)**	**15 (88.2%)**
		OP	2	0	4	4	8
		NSIP	2	0	0	1	4
		Other	1	1	0	0	3
	DILD recovery	All	6	5	2	3	13
Tolerant controls			1	11	16	5	1

The suspected causal drugs in DILD-onset patients and the pharmaceutical products administered to tolerant control patients were categorized into 5 types: DNA-damaging agents (DDAs) including gemcitabine, platinum-based drugs, irinotecan, and other related agents; taxanes including paclitaxel and docetaxel; tyrosine kinase inhibitors (TKIs) including erlotinib, osimertinib, axitinib, bevacizumab, and other related agents; immune checkpoint inhibitors (ICIs) including nivolumab, pembrolizumab, and other related agents; and other pharmaceutical products (other drugs) including mTOR inhibitors and pharmaceutical products for non-cancerous diseases. Patients who received two or more agents (e.g., irinotecan and oxaliplatin) were not counted multiple times within the same group. Data of the DAD and non-DAD groups are shown in bold. DAD: diffuse alveolar damage; OP: organizing pneumonia; NSIP: nonspecific interstitial pneumonia.

Supplementary Table 4. The 55 proteins changed in patients with DAD.

Target	Target Full Name	UniProt	SOMAscan signal (mean $\log_2$ [RFU])						Fold Change (mean $\log_2$ [FC]), vs control			Effect size (*g* value), vs control		
			HV	DAD group	NSIP	OP	Recov	Control	DAD group	OP	NSIP	DAD group	OP	NSIP
Apo-AI	Apolipoprotein A-I	P02647	12.6	11.2	11.9	11.9	12.3	12.4	-1.2	-0.5	-0.5	3.1	1.3	1.3
Kallistatin	Kallistatin	P29622	15.0	13.8	14.4	14.4	14.8	14.9	-1.1	-0.5	-0.5	2.8	1.3	1.2
CAPG	Macrophage-capping protein	P40121	10.4	12.9	11.9	11.4	11.4	11.0	1.9	0.4	0.9	2.2	0.5	1.1
Afamin	Afamin	P43652	14.5	13.4	14.0	13.9	14.3	14.4	-1.0	-0.5	-0.4	2.0	0.9	0.8
Carbonic anhydrase 6	Carbonic anhydrase 6	P23280	12.5	9.4	11.2	10.9	11.6	12.0	-2.6	-1.1	-0.8	2.0	0.9	0.6
PARC	C-C motif chemokine 18	P55774	11.8	13.7	12.8	13.1	12.3	12.1	1.6	1.0	0.7	2.0	1.2	0.8
Stratifin	14-3-3 protein sigma	P31947	13.0	14.5	13.6	13.3	13.4	13.3	1.2	0.1	0.3	2.0	0.1	0.6
IL-1Ra	Interleukin-1 receptor antagonist protein	P18510	11.6	13.4	12.5	12.3	12.2	11.9	1.5	0.3	0.5	2.0	0.6	0.9
HGF	Hepatocyte growth factor	P14210	10.1	11.6	10.6	10.4	10.6	10.4	1.2	0.0	0.3	1.9	0.0	0.5
IGFBP-3	Insulin-like growth factor-binding protein 3	P17936	10.5	9.2	10.0	10.0	10.0	10.2	-1.0	-0.2	-0.2	1.9	0.4	0.5
Cathepsin V	Cathepsin L2	O60911	10.4	9.1	9.4	9.7	9.9	10.1	-1.0	-0.4	-0.7	1.9	0.8	1.2
CYTF	Cystatin-F	O76096	9.5	10.8	9.8	10.0	9.9	9.7	1.1	0.3	0.1	1.9	0.5	0.1
sPLA2	Phospholipase A2, membrane associated	P14555	10.9	13.9	12.3	12.2	12.1	11.6	2.3	0.6	0.7	1.9	0.5	0.6
TNF sR-I	Tumor necrosis factor receptor superfamily member 1A	P19438	9.4	10.9	10.1	10.1	10.2	9.9	1.0	0.2	0.2	1.8	0.4	0.4
SAA	Serum amyloid A-1 protein	P0DJI8	9.6	14.4	12.6	11.6	11.1	10.5	3.9	1.1	2.1	1.8	0.5	0.9

CNDP1	Beta-Ala-His dipeptidase	Q96KN2	12.2	10.7	11.5	11.5	11.8	11.9	-1.3	-0.5	-0.5	1.7	0.7	0.6
MIC-1	Growth/differentiation factor 15	Q99988	9.7	11.8	10.9	10.7	10.9	10.4	1.4	0.3	0.6	1.7	0.3	0.7
TNF sR-II	Tumor necrosis factor receptor superfamily member 1B	P20333	12.8	14.4	13.9	13.7	13.8	13.4	1.0	0.3	0.5	1.7	0.5	0.9
PAPP-A	Pappalysin-1	Q13219	12.8	14.3	14.0	14.0	13.3	13.1	1.3	0.9	0.9	1.6	1.3	1.2
Troponin T	Troponin T, cardiac muscle	P45379	11.0	12.9	12.0	11.7	11.9	11.5	1.3	0.2	0.5	1.6	0.2	0.6
CRP	C-reactive protein	P02741	13.6	17.2	16.6	16.6	15.4	14.7	2.5	1.9	1.9	1.5	1.2	1.2
IL-6	Interleukin-6	P05231	9.6	10.7	9.3	9.5	9.1	9.3	1.4	0.2	0.0	1.5	0.2	0.0
IL-9	Interleukin-9	P15248	8.1	8.9	8.1	8.1	7.8	7.9	1.0	0.2	0.1	1.5	0.5	0.3
Epo	Erythropoietin	P01588	9.8	12.3	11.0	11.4	11.0	10.5	1.8	0.9	0.5	1.5	0.8	0.4
IGFBP-2	Insulin-like growth factor-binding protein 2	P18065	9.2	11.0	9.8	9.9	10.0	9.7	1.3	0.2	0.1	1.5	0.2	0.1
B7-H1	Programmed cell death 1 ligand 1	Q9NZQ7	10.4	11.6	10.6	10.9	10.5	10.5	1.2	0.4	0.1	1.4	0.6	0.2
IP-10	C-X-C motif chemokine 10	P02778	11.3	12.8	12.6	11.9	11.9	11.6	1.1	0.3	1.0	1.4	0.4	1.2
EDAR	Tumor necrosis factor receptor superfamily member EDAR	Q9UNE0	11.8	9.9	10.4	10.2	10.5	11.0	-1.1	-0.8	-0.6	1.4	1.0	0.7
TSP2	Thrombospondin-2	P35442	13.0	15.1	14.5	13.8	14.3	13.8	1.3	0.1	0.7	1.4	0.1	0.7
FLRT3	Leucine-rich repeat transmembrane protein FLRT3	Q9NZU0	11.7	13.1	11.9	12.5	12.1	11.9	1.2	0.5	0.0	1.3	0.6	0.0
Myostatin	Growth/differentiation factor 8	O14793	12.3	11.2	11.9	12.0	12.1	12.2	-1.0	-0.2	-0.3	1.3	0.2	0.4
Stanniocalcin-1	Stanniocalcin-1	P52823	10.6	12.2	11.3	11.3	11.4	11.1	1.2	0.2	0.2	1.3	0.3	0.3

FUT5	Alpha-(1,3)-fucosyltransferase 5	Q11128	11.7	13.4	12.6	13.1	12.6	12.2	1.2	0.9	0.4	1.3	1.0	0.4
MP2K4	Dual specificity mitogen-activated protein kinase kinase 4	P45985	15.9	14.7	15.1	15.3	15.5	15.7	-1.0	-0.3	-0.5	1.3	0.5	0.7
H2B2E	Histone H2B type 2-E	Q16778	14.7	16.4	15.6	15.1	15.6	15.2	1.2	-0.1	0.4	1.3	0.1	0.4
Histone H2A.z	Histone H2A.z	P0C0S5	10.2	12.4	11.5	11.0	11.5	11.0	1.5	0.0	0.6	1.2	0.0	0.5
MIG	C-X-C motif chemokine 9	Q07325	9.4	11.0	10.6	10.2	10.0	9.7	1.3	0.5	0.8	1.2	0.5	0.8
Proteinase-3	Myeloblastin	P24158	11.0	12.5	12.0	12.0	11.8	11.5	1.0	0.5	0.5	1.2	0.6	0.6
Troponin I	Troponin I, fast skeletal muscle	P48788	12.3	13.4	12.5	12.4	12.3	12.3	1.1	0.0	0.2	1.2	0.0	0.2
MIP-3b	C-C motif chemokine 19	Q99731	8.7	10.0	9.3	9.7	9.2	9.0	1.0	0.6	0.3	1.1	0.7	0.4
Osteopontin	Osteopontin	P10451	12.7	14.1	12.7	13.4	13.0	12.9	1.2	0.5	-0.2	1.1	0.6	0.1
vWF	von Willebrand factor	P04275	14.4	16.2	15.4	15.4	15.5	15.0	1.1	0.4	0.4	1.1	0.4	0.4
Ferritin	Ferritin	P02794<br>P02792	13.6	15.8	14.9	15.1	14.8	14.3	1.5	0.8	0.6	1.1	0.6	0.4
PGAM-B	Phosphoglycerate mutase 1	P18669	15.0	12.1	14.5	14.2	13.4	14.1	-2.0	0.1	0.4	1.1	0.1	0.2
BLC	C-X-C motif chemokine 13	O43927	11.3	13.6	12.3	12.2	13.0	12.3	1.3	-0.1	0.0	1.0	0.1	0.0
N-terminal pro-BNP	N-terminal pro-BNP	P16860	11.5	13.7	13.0	12.3	12.9	12.3	1.4	0.0	0.7	1.0	0.0	0.5
LEAP-1	Hepcidin	P81172	13.0	14.3	13.2	13.4	12.9	12.9	1.4	0.5	0.3	1.0	0.3	0.2
ENPP7	Ectonucleotide pyrophosphatase/phosphodiesterase family member 7	Q6UWV6	12.4	11.8	12.8	12.7	13.2	12.9	-1.0	-0.1	0.0	1.0	0.2	0.0
IL-1 R4	Interleukin-1 receptor-like 1	Q01638	11.4	13.8	12.7	12.3	13.1	12.4	1.4	-0.1	0.3	0.9	0.1	0.2

FSH	Follicle stimulating hormone	P01215, P01225	11.0	9.9	10.7	10.9	11.0	11.0	-1.1	0.0	-0.3	0.9	0.0	0.3
HCG	Human Chorionic Gonadotropin	P01215, P01233	11.0	9.5	10.2	10.8	10.5	10.7	-1.3	0.1	-0.5	0.8	0.1	0.3
PF-4	Platelet factor 4	P02776	17.0	14.8	15.6	15.9	15.1	15.9	-1.1	0.0	-0.3	0.8	0.0	0.2
C3b	Complement C3b	P01024	13.3	14.6	14.0	14.9	13.7	13.5	1.1	1.4	0.5	0.7	1.1	0.4
IgE	Immunoglobulin E	P01854	11.4	12.7	12.0	12.2	11.4	11.4	1.3	0.8	0.6	0.7	0.4	0.3
FCG2A	Low affinity immunoglobulin gamma Fc region receptor II-a	P12318	8.5	9.9	9.0	8.7	9.0	8.8	1.1	-0.1	0.2	0.5	0.0	0.1

The 55 proteins showing fold changes (FC) >2 in the DAD group compared to the control were extracted based on the SOMAscan data. HV: healthy volunteer; DAD group: acute-phase patients with DAD or DAD-mixed patterns. Recov: recovery-phase DILD patients; Control: data set from HV and Recov; DAD: diffuse alveolar damage; OP: organizing pneumonia; NSIP: nonspecific interstitial pneumonia.

Supplementary Table 5. Protein candidates that exhibited marked change in patients with DAD.

Change	Target	Full name	UniProt	Fold Change (FC)			Effect size (*g* value)		
				DAD group	OP	NSIP	DAD group	OP	NSIP
Up-regulation	CAPG	Macrophage-capping protein	P40121	3.7	1.4	1.9	2.2	0.5	1.1
	PARC	C-C motif chemokine 18	P55774	3.1	2.0	1.7	2.0	1.2	0.8
	SFN	Stratifin, 14-3-3 protein sigma	P31947	2.3	1.0	1.2	2.0	0.1	0.6
	IL-1Ra	Interleukin-1 receptor antagonist	P18510	2.8	1.2	1.4	2.0	0.6	0.9
	sPLA2	Phospholipase A2, membrane associated	P14555	5.0	1.5	1.7	1.9	0.5	0.6
Down-regulation	CA6	Carbonic anhydrase 6	P23280	0.39	0.89	1.2	2.0	0.9	0.6
	KAL	Kallistatin	P29622	0.45	0.69	0.69	2.8	1.3	1.2
	Apo-AI	Apolipoprotein A-I	P02647	0.42	0.69	0.71	3.1	1.3	1.3

Healthy volunteers and the recovery group were set as comparative control groups. Based on the results of the SOMAscan assay, proteins with fold change (FC) >2.0 or FC <0.5, and an effect size *g* >1.9 in the DAD group (acute-phase patients with DAD or DAD-mixed patterns) were extracted when compared with the control group. DAD: diffuse alveolar damage; OP: organizing pneumonia; NSIP: nonspecific interstitial pneumonia.

Supplementary Table 6. Performance of the in-house SFN ELISA assay system.

Validation item	Performance
Measurement range	0.114 - 30 ng/mL
Minimum required dilution	1-fold
Dilutional linearity	1 : 1 - 1 : 256
Spike-in recovery	within ± 20%
Within run	within ± 20% (accuracy), CV < 15%
Between runs	within ± 20% (accuracy), CV < 15%
Between days	within ± 20% (accuracy), CV < 15%
Selectivity	Not significantly affected by bilirubin C (211 mg/mL) and F (199 mg/mL), hemolytic hemoglobin (4900 mg/dL), chyle (16300 FTU), ascorbic acid (0.2 mg/mL), HAMA (500 mg/mL), rheumatoid factor (500 IU/mL), albumin (50 mg/mL), lipid (1.6 g/dL), or human IgG (28 mg/mL).
Specificity	Not reacted with human 14-3-3 family proteins except for stratifin (100 ng/mL of 14-3-3 α/β, γ, ε, η, ζ, τ/θ, and ζ isoforms)
Stability	Short term stability (stable for at least 72 h at 4°C, 48 h at room temperature, and 6 h at 37°C, and for at least 5 freeze-thaw cycles)

Supplemental Table 7. Blood levels of sPLA2, PARC, KAL and Apo-AI in the Discovery and Validation cohorts.

			Concentration median (range), [*n*]			
			**sPLA2**	**PARC**	**KAL**	**Apo-AI**
**Measurement**		**Matrix**	**plasma**	**plasma**	**serum**	**plasma**
		**Min. Dilution**	**50-fold**	**100-fold**	**5000-fold**	**200-fold**
		**LLoQ (unit)**	**1.6 (ng/mL)**	**3.1 (ng/mL)**	**0.3 (μg/mL)**	**120 (μg/mL)**
HV		Discovery	4.2 (2.6 - 6.1) [11]	24.1 (12.8 - 34.6) [11]	15.1 (9.1 - 21.9) [24]	1040 (817 - 1577) [9]
		Validation	3.3 (2.4 - 5.8) [16]	24.0 (7.7 - 51.5) [16]	14.5 (6.5 - 19.6) [53]	1014 (647 - 1106) [7]
DILD acute	DAD	Discovery	156 (35.5 - 1577.0) [6]	101.4 (30.9 - 157.1) [6]	5.0 (3.0 - 7.5) [6]	321 (237 - 901) [6]
		Validation	80.5 (8.3 - 253.6) [8]	65.6 (35.6 - 121.4) [6]	5.7 (2.7 - 9.9) [11]	506 (199 - 1374) [11]
	DAD-mixed	Discovery	38.8 (4.5 - 81.5) [4]	81.5 (39.7 - 134.0) [4]	5.9 (3.8 - 8.3) [4]	265 (220 - 806) [4]
		Validation	94.0 (16.4 - 179.3) [5]	89.9 (26.5 - 180.8) [5]	6.0 (1.2 - 8.9) [5]	372 (286 - 647) [5]
	OP	Discovery	8.9 (4.5 - 439) [13]	43.4 (17.4 - 655.1) [13]	9.5 (5.3 - 19.5) [13]	868 (211 - 1538) [13]
		Validation	6.4 (2.2 - 284.7) [9]	51.2 (13.0 - 115.3) [8]	9.1 (3.0 - 13.6) [17]	1257 (382 - 2364) [11]
	NSIP	Discovery	11.7 (1.6 - 169.1) [15]	55.5 (11.9 - 270.4) [15]	7.7 (2.8 - 19.0) [15]	977 (378 - 2418) [15]
		Validation	31.7 (2.3 - 208.7) [7]	25.4 (20.9 - 43.3) [3]	9.9 (4.7 - 11.5) [7]	614 (134 - 2336) [7]
	Other	Discovery	8.8 (6.6 - 11.0) [2]	115.4 (102.0 - 128.9) [2]	10.3 (9.6 - 11.0) [2]	811 (468 - 1153) [2]
		Validation	28.0 (7.4 - 35.3) [3]	65.8 [1]	6.6 (3.4 - 13.9) [4]	1008 (477 - 1972) [3]
DILD recovery	All	Discovery	6.8 (2.1 - 371.0) [31]	25.7 (4.0 - 483.8) [31]	12.2 (3.8 - 27.8) [31]	1272 (361 - 4368) [31]
		Validation	6.7 (2.3 - 321.9) [19]	33.7 (13.3 - 96.5) [16]	12.4 (5.1 - 18.7) [23]	972 (259 - 2881) [21]

The matrix used, the minimum dilution factors, the lower limits of quantification (LLoQ), and the concentration (median and range) of each biomarker in healthy volunteers (HV) and DILD patients, as well as the number of samples measured (*n*) in the Discovery and Validation cohorts are indicated. DAD, diffuse alveolar damage; OP, organizing pneumonia; NSIP, nonspecific interstitial pneumonia.

Supplementary Table 8. Serum levels of SFN and known biomarkers in the Combined cohort.

Patient group		Concentration median (range), [*n*]		
		SFN (ng/mL)	KL-6 (U/mL)	SP-D (ng/mL)
HV		0.2 (0.1 - 1.9) [77]	165.8 (105.1 - 408.0) [77]	45.4 (8.6 - 164.2) [77]
DILD acute	DAD	8.5 (2.1 - 48.3) [17]	1136 (316.2 - 5366) [17]	297.9 (82.7 - 1072) [17]
	DAD-mixed	7.9 (1.5 - 20.5) [9]	733.9 (334.1 - 1784) [9]	214.4 (70.5 - 635.1) [9]
	OP	1.3 (0.1 - 24.1) [30]	1020 (150.5 - 4217) [30]	173.8 (24.6 - 1908) [30]
	NSIP	2.1 (0.1 - 43.9) [22]	856.0 (203.0 - 6826) [22]	179.4 (8.6 - 1463) [22]
	Other	2.7 (0.3 - 11.5) [6]	322.8 (228.5 - 532.4) [6]	148.5 (56.2 - 443.8) [6]
DILD recovery	All	0.8 (0.1 - 87.0) [54]	514.6 (112.7 - 18959) [54]	98.2 (8.6 - 436.7) [54]
Tolerant control (no DILD onset)		0.8 (0.1 - 21.2) [31]	343.7 (157.6 - 4413) [31]	59.4 (8.6 - 245.6) [31]
Lung cancer		1.2 (0.1 - 36.0) [58]	369.5 (138.7 - 5936) [58]	69.5 (8.6 - 460.3) [58]
Infectious		0.6 (0.1 - 5.4) [19]	312.6 (118.3 - 1811) [19]	86.7 (8.6 - 493.0) [19]
NTM		0.3 (0.1 - 3.7) [14]	315.9 (185.6 - 1404) [14]	102.2 (8.6 - 429.3) [14]
IIPs		1.3 (0.3 - 18.3) [43]	1311 (189.7 - 9177) [43]	316.8 (20.7 - 766.3) [43]
CTD		1.3 (0.1 - 3.9) [25]	1310 (175.4 - 5593) [25]	122.5 (8.6 - 538.6) [25]
COPD		0.7 (0.1 - 5.4) [15]	242.3 (189.7 - 7762) [15]	72.3 (8.6 - 212.2) [15]
BA		0.3 (0.1 - 0.9) [12]	220.3 (116.8 - 448.8) [12]	51.4 (8.6 - 85.4) [12]

Data of the Combined set are shown. Median values (ranges) of blood levels are shown. [*n*]: number of patients analyzed. DAD: diffuse alveolar damage; OP: organizing pneumonia; NSIP: nonspecific interstitial pneumonia; IIPs: idiopathic interstitial pneumonias; CTD: connective tissue disease; COPD: chronic obstructive pulmonary disease; NTM: nontuberculous mycobacteria; [BA]: bronchial asthma; infection: bacterial and mycotic pneumonia.

Supplementary Table 9. Blood levels of KAL, sPLA2, PARC, Apo-AI in the Combined cohort.

Patient group		Concentration median (range), [*n*]			
		KAL (μg/mL)	sPLA2 (ng/mL)	PARC (ng/mL)	Apo-AI (μg/mL)
HV		14.7 (6.5 - 21.9) [77]	3.9 (2.4 - 6.1) [27]	24.1 (7.7 - 51.5) [27]	1022 (646.6 - 1577) [16]
DILD acute	DAD	5.6 (2.7 - 9.9) [17]	113.2 (8.3 - 1577) [14]	89.6 (30.9 - 157.1) [12]	463.7 (198.8 - 1374) [17]
	DAD-mixed	6.0 (1.2 - 8.9) [9]	70.1 (4.5 - 179.3) [9]	89.9 (26.5 - 180.8) [9]	359.7 (220.4 - 805.7) [9]
	OP	9.3 (3.0 - 19.5) [30]	8.6 (2.2 - 439) [22]	43.4 (13 - 655.1) [21]	930.3 (210.8 - 2364) [24]
	NSIP	8.6 (2.8 - 19.0) [22]	19.9 (1.6 - 208.7) [22]	54.6 (11.9 - 270.4) [18]	765.6 (134.3 - 2418) [22]
	Other	9.3 (3.4 - 13.9) [6]	11 (6.6 - 35.3) [5]	102 (65.8 - 128.9) [3]	1008 (468.2 - 1972) [5]
DILD recovery	All	12.2 (3.8 - 27.8) [54]	6.7 (2.1 - 371) [50]	29.2 (4 - 483.8) [47]	1079 (259.3 - 4368) [52]
Tolerant control (no DILD onset)		13.0 (3.9 - 20.4) [31]	5.5 (3.6 - 623.1) [12]	30.5 (3.5 - 286.4) [9]	932.4 (335.2 - 2893) [20]
Lung cancer		12.8 (3.7 - 21.9) [58]	8 (1.5 - 280.2) [32]	35.4 (19.4 - 1136) [32]	718.4 (267.1 - 1358) [32]
Infectious		6.9 (2.8 - 14.9) [19]	88.3 (1.5 - 3904) [13]	55.7 (21 - 242.7) [13]	660.9 (368.6 - 1139) [17]
NTM		11.8 (5.7 - 18.2) [14]	11.2 (2.6 - 87.4) [12]	37.3 (16.3 - 216.4) [8]	848.2 (437 - 3194) [12]
IIPs		11.3 (5.2 - 19.1) [43]	7.2 (1.7 - 215.7) [20]	82.5 (16.4 - 308.8) [20]	1039 (415.2 - 2289) [40]
CTD		13.8 (2.7 - 22.6) [25]	12 (2.5 - 4026) [19]	74 (18.7 - 876.5) [16]	1144 (377.1 - 3096) [19]
COPD		11.9 (7.2 - 16.2) [15]	10.3 (4.2 - 80.7) [11]	24.1 (5.3 - 119.4) [11]	657.3 (299.1 - 2589) [12]
BA		15.2 (9.4 - 18.6) [12]	8 (3.8 - 15.4) [11]	30.1 (13.7 - 106.8) [11]	918.4 (383.6 - 2113) [11]

Data of the Combined set are shown. Median values (ranges) of blood levels are shown. [*n*]: number of patients analyzed. DAD: diffuse alveolar damage; OP: organizing pneumonia; NSIP: nonspecific interstitial pneumonia; IIPs: idiopathic interstitial pneumonias; CTD: connective tissue disease; COPD: chronic obstructive pulmonary disease; NTM: nontuberculous mycobacteria; [BA]: bronchial asthma; infection: bacterial and mycotic pneumonia.

Supplementary Table 10. Comparison of SFN-positive rates by underlying disease in DILD-onset patients and tolerant control patients.

Patient group		%, SFN positive rate (N, positive/N, analyzed)					
		Lung cancer	Positive subtype	Other tissue cancer	Positive cancer	Non-cancer disease	Positive disease
DILD acute	DAD group	100 (9/9)		90 (10/11)		83 (5/6)	
	non-DAD group	25 (4/16)	1 Ad, 3 SC	20 (5/25)	2 pancreatic, 2 renal, 1 mesothelioma	5.8 (1/17)	1 heart failure
DILD recovery	All	9.1 (1/11)	1 Ad	13 (3/24)	2 pancreatic, 1 esophageal	6.6 (1/15)	1 heart failure
Tolerant controls		10 (3/29)	1 Ad, 1 Ad + Sq, 1 unknown	0 (0/1)		0 (0/1)	

Data of the Combined set are shown. SFN >3.6 ng/mL was defined as positive. SFN-positive rates (number of samples tested positive per number of samples measured) in DILD patients and tolerant control patients who had any of the following conditions as underlying diseases were examined: lung cancer, other cancers, and non-cancer conditions. For patients who were not in the DAD group (OP+NSIP+others), the tissue types of lung cancer that showed positive results as well as the details of the diseases are shown. Ad: adenocarcinoma; SC: small-cell carcinoma; Sq: squamous cell carcinoma. DAD: diffuse alveolar damage; OP: organizing pneumonia; NSIP: nonspecific interstitial pneumonia.

Supplementary Table 11. Comparison of SFN-positive rates by medication in DILD-onset patients and tolerant control patients.

Patient group		%, SFN positive rate (N, positive/N, analyzed)				
		DDAs	Taxanes	TKIs	ICIs	Other drugs
DILD acute	DAD group	88（7/8）	78 (7/9)	100（5/5）	100 (4/4)	100 (6/6)
	non-DAD group	24 (4/17)	20 (2/10)	17 (1/6)	22 (2/9)	14 (4/29)
DILD recovery	All	12 (2/17)	19 (3/16)	0 (0/4)	0 (0/6)	7.4 (2/27)
Tolerant control (no DILD onset)		0 (0/1)	18 (2/11)	0 (0/16)	40 (2/5)	0 (0/1)

Data of the Combined set are shown. The suspected causative drugs in DILD patients as well as the pharmaceutical products administered to tolerant control patients were categorized by type. SFN positive rates were compared between patients who received different pharmaceutical products. SFN >3.6 ng/mL was defined as positive. DDAs: DNA-damaging agents; taxane: taxanes; TKIs: tyrosine kinase inhibitors (e.g., EGFR-TKI and EGFR inhibitors); ICIs: immune checkpoint inhibitors; DAD: diffuse alveolar damage; OP: organizing pneumonia; NSIP: nonspecific interstitial pneumonia.
