# Supplementary material for: Stratifin as a novel diagnostic biomarker in serum for diffuse alveolar damage
Source: Nat Commun. 2022 Oct 4;13:5854. doi: 10.1038/s41467-022-33160-9 (PMC9532442; doi:10.1038/s41467-022-33160-9)
Supplement: Supplementary file 2 — Reporting Summary [file 41467_2022_33160_MOESM2_ESM.pdf]

nature portfolio

Corresponding author(s): Yoshiro Saito

Last updated by author(s): Aug 24, 2022

Reporting Summary

Nature Portfolio wishes to improve the reproducibility of the work that we publish. This form provides structure for consistency and transparency in reporting. For further information on Nature Portfolio policies, see our Editorial Policies and the Editorial Policy Checklist.

Statistics

For all statistical analyses, confirm that the following items are present in the figure legend, table legend, main text, or Methods section.

n/a	Confirmed	
☐	☒	The exact sample size (n) for each experimental group/condition, given as a discrete number and unit of measurement
☐	☒	A statement on whether measurements were taken from distinct samples or whether the same sample was measured repeatedly
☐	☒	The statistical test(s) used AND whether they are one- or two-sided<br>*Only common tests should be described solely by name; describe more complex techniques in the Methods section.*
☐	☒	A description of all covariates tested
☐	☒	A description of any assumptions or corrections, such as tests of normality and adjustment for multiple comparisons
☐	☒	A full description of the statistical parameters including central tendency (e.g. means) or other basic estimates (e.g. regression coefficient) AND variation (e.g. standard deviation) or associated estimates of uncertainty (e.g. confidence intervals)
☐	☒	For null hypothesis testing, the test statistic (e.g. F, t, r) with confidence intervals, effect sizes, degrees of freedom and P value noted<br>*Give P values as exact values whenever suitable.*
☒	☐	For Bayesian analysis, information on the choice of priors and Markov chain Monte Carlo settings
☒	☐	For hierarchical and complex designs, identification of the appropriate level for tests and full reporting of outcomes
☐	☒	Estimates of effect sizes (e.g. Cohen's d, Pearson's r), indicating how they were calculated

Our web collection on statistics for biologists contains articles on many of the points above.

Software and code

Policy information about availability of computer code

Data collection	Clinical data was collected as anonymized clinical information using a case report form, and then managed using Microsoft Excel (Microsoft Office Home and Buisiness 2019).
Data analysis	All data were analyzed using GraphPad PRISM (version 8.4.3; GraphPad Software, San Diego, CA, USA) and Microsoft Excel (Microsoft Office Home and Buisiness 2019).

For manuscripts utilizing custom algorithms or software that are central to the research but not yet described in published literature, software must be made available to editors and reviewers. We strongly encourage code deposition in a community repository (e.g. GitHub). See the Nature Portfolio guidelines for submitting code & software for further information.

Data

Policy information about availability of data

All manuscripts must include a data availability statement. This statement should provide the following information, where applicable:

- Accession codes, unique identifiers, or web links for publicly available datasets
- A description of any restrictions on data availability
- For clinical datasets or third party data, please ensure that the statement adheres to our policy

The data that support the findings of this study including clinical information, and SOMAscan and ELISA data are available within the paper and its Supplementary Information. The source data underlying all figures and supplementary figures are provided as a Source Data file. The immunohistochemistry images for SFN expression in normal tissues referenced in this study are available in the Human Protein Atlas database (https://www.proteinatlas.org/ENSG00000175793-SFN/tissue).

Field-specific reporting

Please select the one below that is the best fit for your research. If you are not sure, read the appropriate sections before making your selection.

☒ Life sciences ☐ Behavioural & social sciences ☐ Ecological, evolutionary & environmental sciences

For a reference copy of the document with all sections, see nature.com/documents/nr-reporting-summary-flat.pdf

Life sciences study design

All studies must disclose on these points even when the disclosure is negative.

Sample size	The sample size was determined based on the results of the power calculation. For example, the number of "cases" and "controls" required for the analysis was calculated to be 11 and 21, respectively, when a power analysis is conducted using the following criteria: Mann-Whitney (2 groups), α = 0.05, power 80%, effect size = 1.0", number of controls: 2-fold of cases. In this study, we started the study for biomarker discovery when we collected 10 and 30 cases in the DAD and non-DAD groups, respectively, and then searched for proteins with an effect size Hedge's "g >1.9" in the acute diffused alveolar damage (DAD) group (n=10) compared to the control group (all recovery group [n=31] and healthy volunteer [n=24]). The identified protein candidates were validated using a larger number of samples in an independent cohort. We believe that they are considered to be sufficient sample sizes.
Data exclusions	None of the obtained data was excluded.
Replication	All experiments were conducted at least three times, and with similar results reproduced. The findings of the biomarker levels was validated using an independent cohort.
Randomization	This is a observational case-control study. The Discovery cohort of patients with DILD (drug-induced lung disease) who were enrolled in the early phase (April 2015 to November 2016) of this study, and validated the results using a "Validation cohort" consisting of subsequently enrolled DILD patients (December 2016 to May 2019).
Blinding	The blinding was not completely applied for patients' recruitment in this study, because this study was observational -no intervention and exploratory in character. The technicians performing the biomarker assays and immunohistochemistry for human samples were blinded to patient characteristics and diagnoses. The investigators were not blinded in order to perform analyses on comparing different clinical parameters to determine their biological relevance. Also, blinding was not applicable to the in vitro experiments, because the investigators needed to rank and load (ELISA and Western blot) the samples based on the treatment information.

Reporting for specific materials, systems and methods

We require information from authors about some types of materials, experimental systems and methods used in many studies. Here, indicate whether each material, system or method listed is relevant to your study. If you are not sure if a list item applies to your research, read the appropriate section before selecting a response.

Materials & experimental systems

n/a	Involved in the study	
☐	☒	Antibodies
☐	☒	Eukaryotic cell lines
☒	☐	Palaeontology and archaeology
☒	☐	Animals and other organisms
☐	☒	Human research participants
☒	☐	Clinical data
☒	☐	Dual use research of concern

Methods

n/a	Involved in the study	
☒	☐	ChIP-seq
☒	☐	Flow cytometry
☒	☐	MRI-based neuroimaging

Antibodies

Antibodies used

(1) Anti-14-3-3σ SFN, mouse mAb (clone CS112 2A8), Merck (human), Cat#05-632/Lot#3152578
(2) Anti-SFN, mouse mAb (clone 3C3) , Sigma Aldrich (human), Cat#WH0002810M1/Lot#J1221-3C3
(3) Anti-SFN rabbit pAb, Atlas antibodies (human), Cat#HPA011105/Lot#A69410
(4) Anti-p53 mAb (clone DO-7), Invitrogen (human), Cat#MA5-12557/LotUJ290170
(5) Anti-p21 Waf1/Cip1 rabbit mAb (clone 12D1), Cell Signaling Technology, Cat#2947/Lot#11
(6) Anti-beta Actin Rabbit pAb, Proteintech (human), Cat#20536-1-AP /Lot (barcode) #00079207
(7) Anti-phospho-p53 (Ser15) rabbit pAb, Cell Signaling, Cat#9284/Lot#21
(8) Anti-phospho-p53 (Ser392) rabbit pAb, Cell Signaling, Cat#9281/Lot#7
(9) Anti-MDM2, rabbit mAb (clone D1V2Z), Cell Signalling, Cat#/86934/Lot#2
(10) Anti-alpha-tubulin, mouse mAb (clone DM1A), Cell Signaling, Cat#3873/Lot#16
(11) Anti-lamin b1, mouse mAb (clone 3C10G12), Proteintech, Cat#66095-1-Ig/Lot#10019102

(12) HRP-linked anti-mouse IgG, Cell Signaling, Cat#7076/Lot#36
(13) HRP-linked anti-rabbit IgG, Cell Signaling, Cat#7074/Lot#30

Validation	(1) Anti 14-3-3σ SFN mAb (clone CS112 2A8) was validated for immunoprecipitation and Western blot (https://www.merckmillipore.com/JP/ja/product/Anti-14-3-3-Antibody-clone-CS112-2A8,MM_NF-05-632).<br>(2) Anti-SFN mAb (clone 3C3) was validated for immunohistochemistry and Western blot (https://www.sigmaaldrich.com/JP/en/product/sigma/wh0002810m1).<br>(3) Anti SFN rabbit pAb was developed and validated Human Protein Atlas project for immunohistochemistry and Western blot (https://www.sigmaaldrich.com/JP/en/product/sigma/hpa011105)<br>(4) Anti-p53 mAb (clone DO-7) was validated by immunohistochemistry and Western blot (https://www.thermofisher.com/antibody/product/p53-Antibody-clone-DO-7-Monoclonal/MA5-12557)<br>(5) Anti-p21 Waf1/Cip1 rabbit mAb (clone 12D1) was validated by Western blot (https://www.cellsignal.jp/products/primary-antibodies/p21-waf1-cip1-12d1-rabbit-mab/2947)<br>(6) Anti-beta Actin Rabbit pAb was validated by Western blot (https://www.ptglab.co.jp/products/ACTB-Antibody-20536-1-AP.htm).<br>(7) Anti-phospho-p53 (Ser15) rabbit pAb was validated by Western blot (https://www.cellsignal.jp/products/primary-antibodies/phospho-p53-ser15-antibody/9284)<br>(8) Anti-phospho-p53 (Ser392) rabbit pAb was validated by Western blot (https://www.cellsignal.jp/products/primary-antibodies/phospho-p53-ser392-antibody/9281)<br>(9) Anti-MDM2 rabbit mAb (clone D1V2Z) was validated by Western blot (https://www.ptglab.co.jp/products/LMNB1-Antibody-66095-1-Ig.htm)<br>(10) Anti-alpha-tubulin mouse mAb (clone DM1A) was validated by Western blot (https://www.cellsignal.jp/products/primary-antibodies/a-tubulin-dm1a-mouse-mab/3873)<br>(11) Anti-lamin b1 mouse mAb (clone 3C10G12) was validated by Western blot (https://www.ptglab.co.jp/products/LMNB1-Antibody-66095-1-Ig.htm)<br>(12) Anti-mouse IgG, HRP-linked antibody was thoroughly validated with the CST primary antibodies to ensure that they work optimally with Western immunoblotting protocols and provide accurate and reproducible results (https://www.cellsignal.jp/products/secondary-antibodies/anti-mouse-igg-hrp-linked-antibody/7076).<br>(13) Anti-rabbit IgG, HRP-linked antibody was validated with the CST primary antibodies to ensure that they work optimally with Western immunoblotting protocols and provide accurate and reproducible results (https://www.cellsignal.jp/products/secondary-antibodies/anti-rabbit-igg-hrp-linked-antibody/7074).<br><br>The ELISA assay method for stratifin using the antibodies (1) and (2) was analytically validated with reference to the Japanese Guidelines on Bioanalytical Method Validation (Ligand Binding Assay) in Pharmaceutical Development (https://www.pmda.go.jp/files/000206208.pdf). This result was summarized in Supplementary Table 6.

Eukaryotic cell lines

Policy information about cell lines

Cell line source(s)	A549 cell line was purchased from JCRB Cell Bank (Cat#JCRB0076).<br>Primary human small airway epithelial cells (manufacture, Lifeline cell technology) were purchased from Kurabo (Cat#KH-4209(FC-0016)/Lot#01925) for this project. The primary cells were derived from a fully informed consented donor (https://www.lifelinecelltech.com/knowledge-base/ethical-and-legal-standards/).
Authentication	The A549 cells were used with less than 8 passages after purchased from the JCRB cell bank, operated by National Institute of Biomedical Innovation, Health and Nutrition of Japan (https://cellbank.nibiohn.go.jp/english/). The cells were authorized by JCRB cell bank using DNA profile (short tandem repeats).
Mycoplasma contamination	Mycoplasma was not detected when the cells were purchased from the JCRB cell bank and Kurabo.
Commonly misidentified lines<br>(See ICLAC register)	No commonly misidentified cell line was used.

Human research participants

Policy information about studies involving human research participants

Population characteristics	There were two cohorts and controls:<br><br>A) Discovery cohort<br>Samples were collected at the acute and recovery phases from consecutively recruited DILD-onset patients from April 2015 to November 2016 and samples from age-matched healthy volunteers.<br>- DAD group (DILD patients with the DAD or DAD-mixed patterns), consisting 8 males and 2 females (mean age 71 years).<br>- non-DAD group (DILD patients with the non-DAD patterns), consisting 17 males and 13 females (mean age 61.5 years).<br>- healthy volunteers, consisting12 males and 12 female (mean age: 61 years)<br><br>B) Validation cohort<br>Samples were collected from consecutively recruited DILD-onset patients following the Discovery cohort from December 2016 to May 2019 and the healthy volunteers that were not included in the Discovery cohort.<br>- DAD group, consisting 13 males and 3 females (mean age 69 years).<br>- non-DAD group, consisting 13 males and 15 females (mean age 69 years).<br>- healthy volunteers, consisting 20 males and 33 female (mean age 34 years [range 25-64])<br><br>C) Controls

Samples was collected throughout the entire study period (between April 2015 and May 2019).
- Tolerant controls (no DILD onset), consisting 19 males and 12 females (mean age 69 years).
- Disease controls (7 lung diseases) , consisting 122 males and 64 females, (mean age differed across each disease group between 60 and 75).

The detailed clinical information of the cohorts are described in Table 1, Supplementary Tables 1-3 and Supplementary Data 1.

Recruitment

This study contents was explained to the patients (with DILD, tolerant (no onset of DILD), and 7 lung diseases) who fulfill the following inclusion criteria, and healthy volunteers. And then obtaining informed consents.

-DILD: Included are patients with DILD diagnosed according to the Japanese diagnostic criteria by the respiratory specialists (by the Japanese respiratory society), as follows: 1) history of ingestion of a drug that is known to induce lung injury, 2) appearance of clinical manifestations after drug administration, 3) improvement of clinical manifestations after drug discontinuation, 4) exacerbation of clinical manifestations after resuming drug administration (challenge testing), and 5) exclusion of other causes of the clinical manifestations. The criterion 4) is not used due to ethical issues. Excluded are patients who are clearly non-DILD, such as viral infections, bacterial infections, and exacerbated cases of idiopathic interstitial pneumonia, and other patients who the doctor deems unsuitable for this study.

-Tolerant controls: Patients who took similar drugs with the DILD patients, but have not developed DILD over the course of at least three months, and who have no history of lung damage.

-Disease controls: Patients with the 7 types of diseases (The diagnostic criteria for each disease conform to the diagnostic criteria used by academic societies in Japan).

-Healthy volunteers: 1) Japanese (self-declaration that all family members up to grandparents are Japanese) diagnosed as healthy by their physician, 2) fasted for at least 14 h before blood collection (only drinking water is permitted), 3) not consuming any of drugs for at least 1 week, and 4) with a normal body mass index (18.5≤ BMI <25); 5) female individuals menstruating are excluded.

Recruitment of patients was performed based on the judgment of the physicians in four hospitals. The samples were collected in a uniform manner. The samples of DILD patients were divided into the Discovery and Validation cohorts based on the recruitment periods, and analyzed for biomarker level in batches. There is no obvious self-selection bias. However, it was a retrospective study and the number of patients enrolled was small, and thus we cannot completely rule out a potential bias in the selection of patients and possibility of overestimating results.

Ethics oversight

Human Ethics approval was obtained for this study from each research ethics review committee of the participating institutes i.e.), Shinshu University, Nippon Medical School, Chiba University, and Hiroshima University, the National Institute of Health Sciences, the Kihara Foundation, Astellas Pharma Inc., and Daiichi Sankyo Company. Informed consent was obtained from each participant for this study.

Note that full information on the approval of the study protocol must also be provided in the manuscript.
