# Supplementary material for: Stratifin as a novel diagnostic biomarker in serum for diffuse alveolar damage
Source: Nat Commun. 2022 Oct 4;13:5854. doi: 10.1038/s41467-022-33160-9 (PMC9532442; doi:10.1038/s41467-022-33160-9)
Supplement: Supplementary file 3 — Description of Additional Supplementary Files [file 41467_2022_33160_MOESM3_ESM.pdf]

Title: Supplementary Data 1.

Description: Clinical information of the all subjects. Clinical information of healthy volunteers, DILD patients, tolerant controls and disease controls analysed in this study are shown. ND: not determined.

Title: Supplementary Data 2.

Description: The SOMAscan proteomic data of 1310 target proteins in plasma samples from subjects in the Discovery cohort.

Title: Supplementary Data 3.

Description: Blood levels of biomarker protein candidates and SP-D and KL-6 measured using samples from subjects in the Discovery and Validation cohorts, and tolerant and disease controls. ND: not determined
